# Comedonal Plaques With Scarring Alopecia on the Scalp: A Case Report

**DOI:** 10.7759/cureus.80278

**Published:** 2025-03-09

**Authors:** Apoorva Sharma, Meghana K B, Debajyoti Chatterjee, Muthu Sendhil Kumaran

**Affiliations:** 1 Dermatology, Postgraduate Institute of Medical Education and Research, Chandigarh, IND; 2 Histopathology, Postgraduate Institute of Medical Education and Research, Chandigarh, IND

**Keywords:** comedo, cutaneous lupus erythematosus, scalp lesions, scar appearance, scarring alopecia

## Abstract

Comedonal discoid lupus erythematosus (DLE) is a rare variant of chronic cutaneous lupus erythematosus, often posing diagnostic challenges due to its atypical acneiform presentation. We report a case of a 52-year-old male presenting with alopecia and hypertrophic plaques on the scalp, characterized by follicular plugging, open comedones, and dermatoscopic findings of telangiectasias, scattered pigmentation, and follicular loss. Histopathology revealed hallmark features of DLE, including basal layer liquefaction and perifollicular lymphocytic infiltration. Anti-nuclear antibody testing and systemic lupus erythematosus (SLE) workup were negative. Despite its rarity, early recognition of comedonal DLE is critical to prevent scarring alopecia and ensure timely treatment, which includes strict photoprotection, topical corticosteroids, and systemic hydroxychloroquine. This report underscores the importance of considering DLE in cases of refractory acneiform lesions with atypical clinical features.

## Introduction

Discoid lupus erythematosus (DLE) is the most common variant of chronic cutaneous lupus erythematosus (CCLE), characterized by inflammatory skin lesions that can progress to atrophic scarring. It typically presents as erythematous plaques with hyperkeratosis, follicular plugging, and telangiectasias, predominantly affecting the face, scalp, and other sun-exposed areas. While classic DLE is well-documented, rare presentations like comedonal DLE are infrequently reported, complicating early diagnosis and management.

Comedonal DLE, an unusual variant, mimics acne vulgaris due to the presence of open comedones and punctate scars, often leading to delayed recognition. Its pathogenesis, though not fully understood, may involve exaggerated follicular hyperkeratosis and mononuclear infiltration of pilosebaceous units. This overlap with other dermatologic conditions, such as acne vulgaris, nevus comedonicus, or folliculotropic mycosis fungoides (FMF), necessitates a high index of suspicion and thorough evaluation.

In addition to its diagnostic challenges, comedonal DLE holds clinical significance due to its potential for scarring alopecia and association with systemic lupus erythematosus (SLE) in a subset of patients. Prompt diagnosis and intervention can mitigate disease progression, improve outcomes, and minimize complications.

## Case presentation

**Figure 1:**
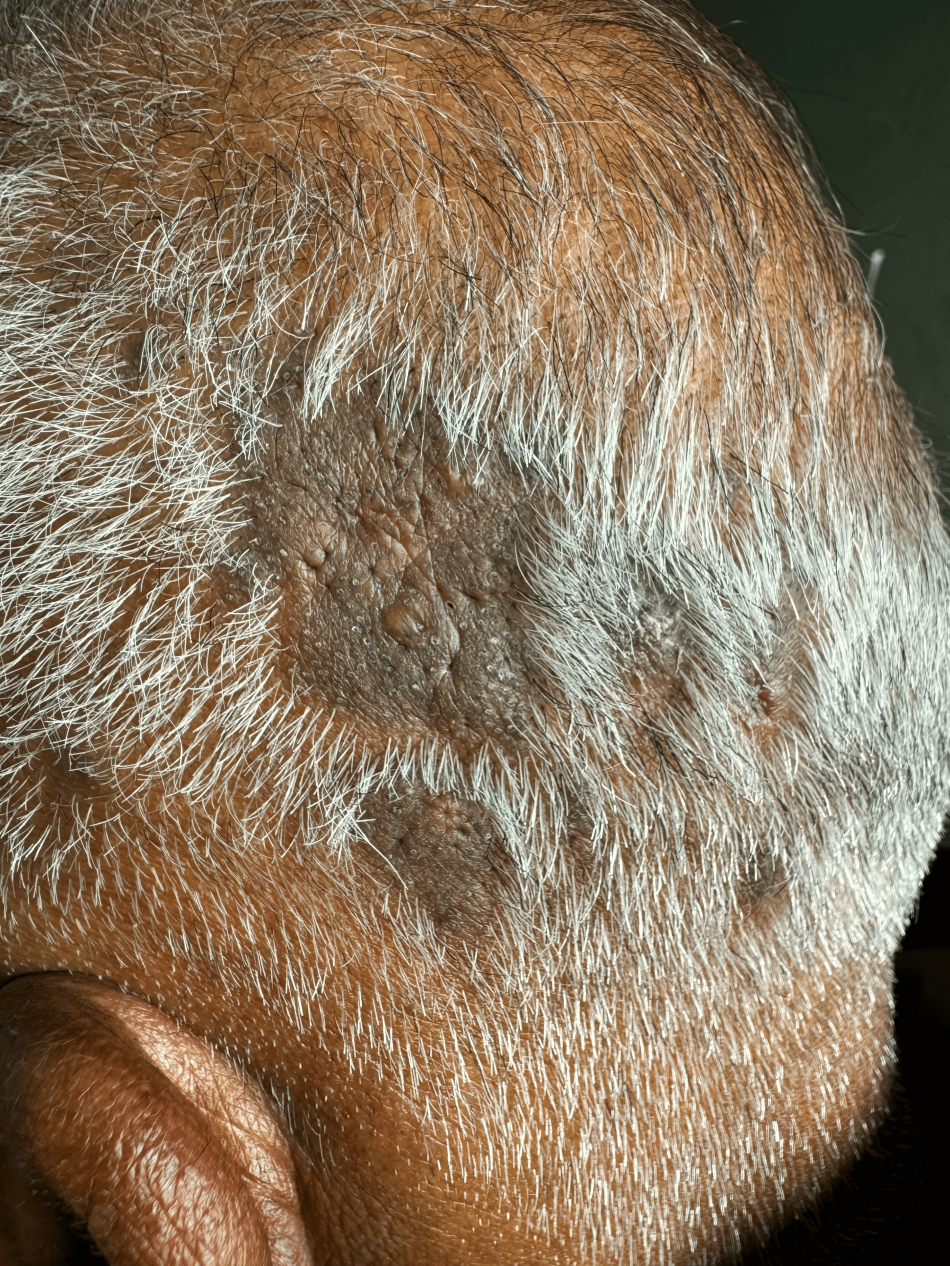
Multiple patches of alopecia with well-defined hypertrophic plaques of varying sizes with minimal scaling, follicular plugs, and a few open comedones overlying the plaque

**Figure 2:**
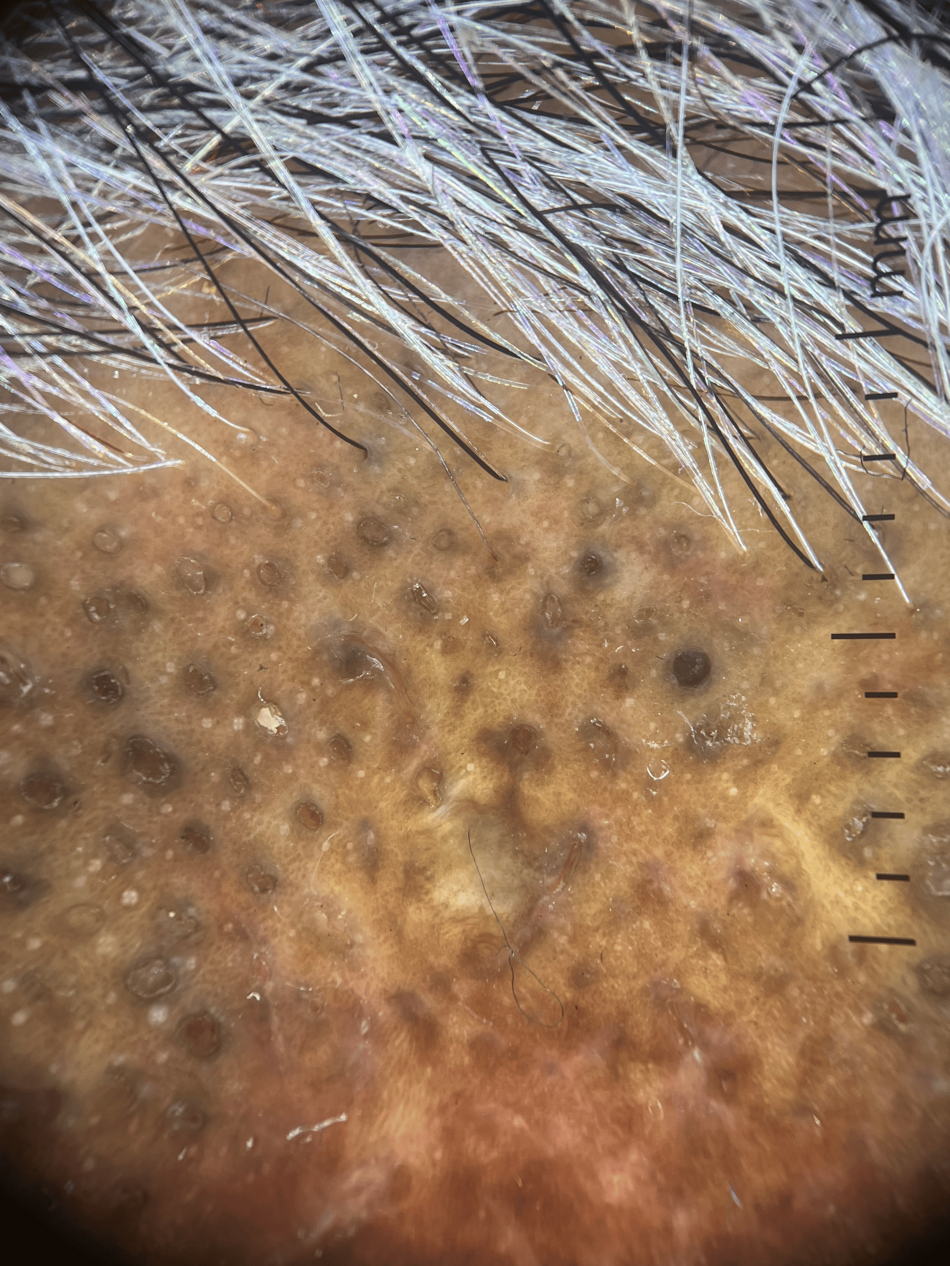
Dermatoscopy revealing white patchy scales, follicular plugging, a few areas of telangiectasias with scattered pigmentation, and areas of loss of follicular openings (DermLite™ DL4; polarized; x10 polarized mode)

**Figure 3:**
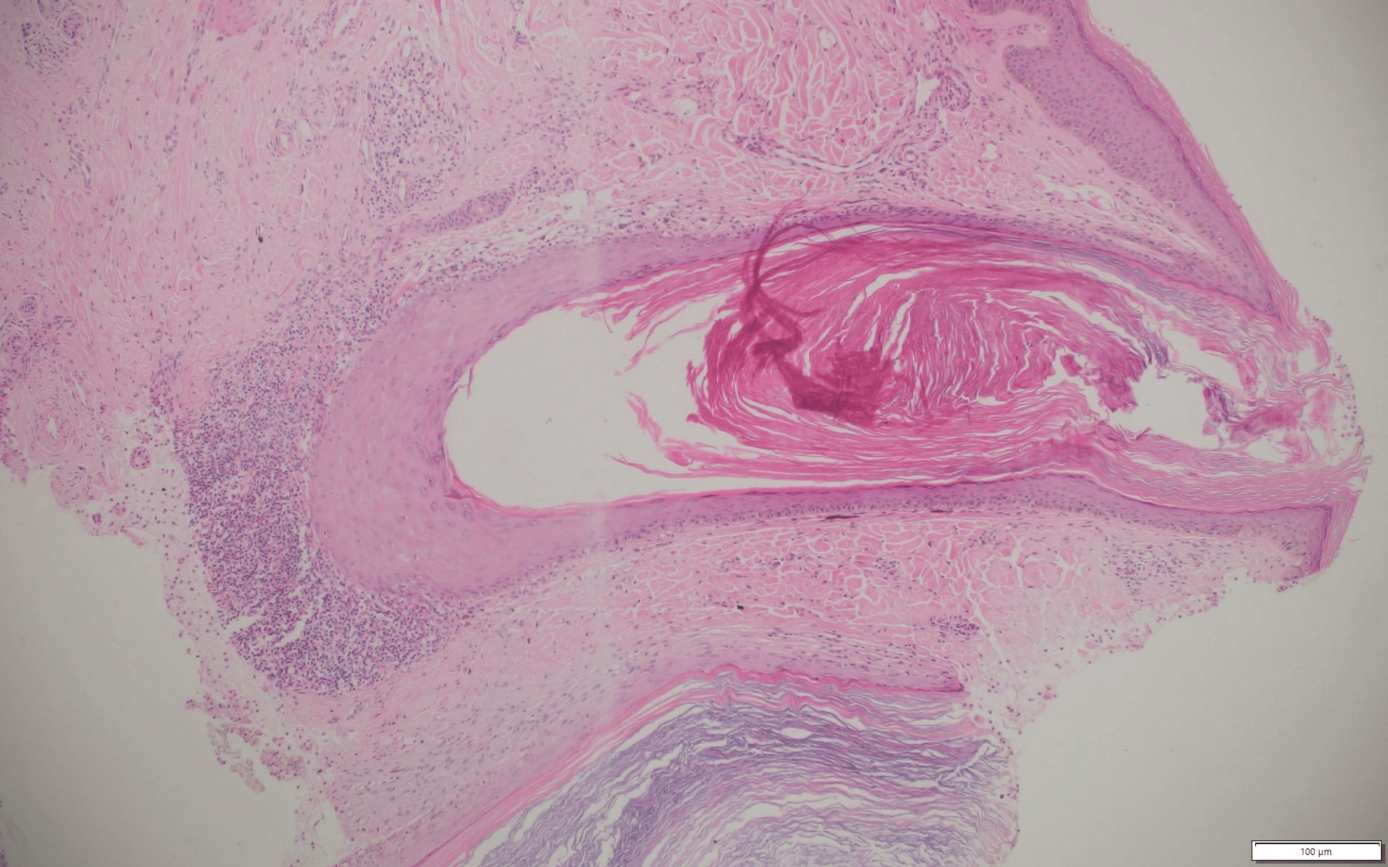
Histopathology showing mild acanthosis in the epidermis with focal basement membrane thickening and liquefaction degeneration of the basal cell layer. Dilated follicular infundibula with perifollicular inflammation (hematoxylin and eosin, 40x)

A 52-year-old male presented with patches of hair loss predominantly over the parietal and occipital parts of the scalp for six months. These lesions initially started as discrete papules, which eventually conglomerated to form plaques with scarring alopecia. The lesions were asymptomatic except for mild occasional itching. Cutaneous examination revealed multiple patches of alopecia with well-defined hypertrophic plaques of varying sizes with minimal scaling, follicular plugs, and a few open comedones overlying the plaque (Figure 1). Dermatoscopy revealed white patchy scales, follicular plugging, a few areas of telangiectasias with scattered pigmentation, and areas of loss of follicular openings (Figure 2). Histopathology showed mild acanthosis in the epidermis, focal basement membrane thickening, and liquefaction degeneration of the basal cell layer. Dilated follicular infundibulum with peri-follicular and peri-eccrine lymphocytic infiltrate with involvement of interfollicular area. Deep dermal inflammation along with melanin incontinence is seen (Figure 3). A diagnosis of comedonal DLE was made based on clinical, dermatoscopic, and histopathological findings. Anti-nuclear antibody (ANA) was negative, and there were no clinical or laboratory signs of SLE.

## Discussion

Cutaneous lupus erythematosus is classified into acute, subacute, and chronic types, each with a different morphology. CCLE, particularly DLE, is defined by pink-to-violaceous plaques with hyperkeratosis and follicular plugging. These lesions often lead to atrophy, scarring, hyperpigmentation, and telangiectasias. DLE on hair-bearing skin can cause cicatricial alopecia, and it is most common on the face, ears, scalp, and sun-exposed areas. Comedonal DLE is a very rare but documented variant of cutaneous lupus erythematosus. According to the literature, this condition is more frequent among women aged between the third and fourth decades of life, with an average age of 38.9 years [1]. Similar to DLE, this variant also favors the face, ears, scalp, and sun-exposed areas. The clinical features encompass comedones, erythematous papules, and punctate scars, mainly in sun-exposed areas. Differentiating features of this condition from other acneiform eruptions may include the coexistence of classic DLE lesions, the merging of lesions into infiltrated plaques, perilesional erythema, and the presence of telangiectasias. Pruritus is a common symptom.

Given that DLE can exhibit a Koebner response, there is a possibility of an isomorphic DLE response in a lesion otherwise characteristic of acne vulgaris. Some suggest that acneiform lesions may result from the mononuclear cell infiltration of the pilosebaceous units [2]. Both DLE and acne vulgaris share follicular hyperkeratosis in their pathology, so it is plausible that an exaggeration of this intrinsic hyperkeratosis in DLE could manifest clinically and histologically as a comedonal process. Furthermore, follicular mycosis fungoides with prominent comedonal lesions provide precedent for additional T-cell lymphocyte-rich reactions that produce a follicular cystic pattern of hyperkeratosis resembling a comedone [3].

Other benign dermatological disorders such as nevus comedonicus, Favre-Racouchot disease, agminate lichen follicularis with cysts and comedones, and especially acne vulgaris are examples of differential diagnoses. The other most serious differential diagnosis for itchy scarring alopecia with comedones is FMF [4]. However, unlike these benign conditions, CCLE carries a substantial destructive risk, leading to atrophic scarring and even mutilations if left untreated, as observed in our case. Furthermore, 31% of those with comedonal DLE also have concurrent systemic erythematous lupus, highlighting the substantial morbidity linked to treatment and diagnosis delay [5]. In a case series published by Droesch and Magro, three out of nine patients had positive ANA tests, and two of them had systemic involvement and met the criteria of SLE [6]. However, it is variable across different case reports. Our patient was evaluated for systemic involvement, which returned negative.

Management of this particular form of DLE is challenging and consists of strict photoprotection. A combination of topical and/or intralesional corticosteroids and retinoids such as tretinoin and tazarotene may help improve the condition. However, systemic therapy is often necessary, with hydroxychloroquine (HCQ) considered the primary choice.

Due to its rarity and limited awareness of comedonal DLE among dermatologists, the diagnosis may be delayed, adversely affecting the quality of life. Recognizing this as a possible differential diagnosis in acneiform conditions, particularly when atypical manifestations and a suboptimal response to conventional treatments are present, is crucial. Timely diagnosis and intervention have the potential to alleviate morbidity, diagnose SLE at an early stage, and also mitigate the risk of scarring alopecia.

## Conclusions

This case report highlights a case of comedonal DLE in a middle-aged male, detailing its clinical, dermatoscopic, and histopathological features to aid in the early identification and appropriate management of this rare dermatological entity. This condition is often misdiagnosed, leading to a delay in diagnosis. This detailed exploration of comedonal DLE contributes to the existing literature and aids in refining clinical approaches to diagnosing and managing this uncommon dermatological entity.
